# Culture‐Negative Septic Pulmonary Embolism Secondary to Lower‐Limb Cellulitis Following Recent Dengue Fever Complicated by Hydropneumothorax: A Case Report

**DOI:** 10.1002/ccr3.73513

**Published:** 2026-09-11

**Authors:** Faiza Arif, Mariyah Saqib Qureshi, Sajjad Khan, Malaika Usman, Umama Rehman, Aiyna Usman, Bilal Wazir Khan, Kashif Yousaf

**Affiliations:** ^1^ Department of Internal Medicine Khyber Teaching Hospital Peshawar Pakistan; ^2^ Khyber Medical College Peshawar Pakistan; ^3^ Medical Faculty Jalal‐Abad State University Named After B. Osmonov Jalal‐Abad Kyrgyzstan

**Keywords:** bacterial coinfection, case report, cavitary pulmonary nodules, cellulitis, culture‐negative infection, dengue fever, hydropneumothorax, septic pulmonary embolism

## Abstract

Secondary bacterial infection and septic pulmonary embolism should be considered in patients recovering from dengue fever who develop focal soft‐tissue infection, respiratory symptoms, or markedly elevated inflammatory markers. Characteristic radiological findings may establish the diagnosis even when blood cultures are negative and infective endocarditis is absent.

## 1 Introduction

Septic pulmonary embolism (SPE) is an uncommon, life‐threatening type of pulmonary embolism resulting from infected thrombi that block the pulmonary arteries, thereby causing infarction and abscesses in the lung. It is associated with largely nonspecific clinical manifestations which often result in a delay in diagnosis. Consequently, diagnosis often relies on characteristic radiological findings together with identification of predisposing risk factors. Chest CT typically demonstrates multiple peripheral pulmonary nodules or wedge‐shaped infiltrates, often with cavitation and occasional pleural effusions. The likely sources of infection can be identified, with Lemierre's syndrome, intravenous drug use, indwelling catheters or prosthetic devices, and skin or soft tissue infections being the most common predisposing factors. *Staphylococcus aureus* is one of the most frequently identified pathogens. Both methicillin‐sensitive and methicillin‐resistant strains are often reported in SPE series [1, 2].

Dengue fever is the most important arthropod‐borne viral disease in the tropics. It can be a mild febrile illness, or it can be fatal hemorrhagic fever and shock. Co‐infection with bacteria in dengue is rare but increasingly reported. Clinically, persistent or prolonged fever is an important clue to possible bacterial coinfection. Lee et al. reported that about 5.5% of their dengue hemorrhagic fever patients had concomitant bacteremia. Moreover, patients with dengue who had fever for more than 5 days were at significantly higher risk of having concurrent bacteremia. These bacterial co‐infections seriously affect the clinical course and have been associated with increased morbidity and mortality [3, 4, 5, 6].

While bacterial coinfections of dengue are becoming increasingly recognized, septic pulmonary embolism secondary to cellulitis after dengue infection appears to be very rare, with only a few reports in the literature. Here, we report a case of culture‐negative septic pulmonary embolism most likely secondary to left lower‐limb cellulitis occurring during recovery from dengue fever and complicated by hydropneumothorax.

## 2 Patient Information

A 22‐year‐old man with a 2‐day history of progressive swelling of the left lower limb with associated exertional dyspnoea presented to the emergency department. He had been diagnosed with dengue fever (NS1 antigen positive) 7 days prior, which had led to complications of thrombocytopenia and leukopenia. He was managed as an outpatient supportively.

Four days prior to his admission, he had a small episode of spontaneous epistaxis. He denied chest pain, orthopnea, fever, or any other systemic symptoms on presentation. The only past medical history of note was generalized anxiety disorder for which he was on clonazepam and paroxetine. He was not on any immunosuppressants. He denied a history of deep vein thrombosis, chronic leg swelling, cellulitis, or other significant infections or surgeries in the past. He had never been hospitalized for his current illness and had been in his usual state of health prior to this event.

He denied any personal history of tuberculosis or other chronic diseases or any known exposure to tuberculosis. His family history was significant for pulmonary tuberculosis in a first‐degree relative. He had no history of tobacco, alcohol, or illicit drug use.

## 3 Clinical Findings and Examination

A 22‐year‐old man sitting in bed with a visibly swollen left leg which was elevated. On examination, the patient appeared pale. He was afebrile, with a heart rate of 110 beats/min, blood pressure of 100/60 mmHg, respiratory rate of 18 breaths/min, and oxygen saturation of 98% on room air. There were no stigmata of chronic disease on general examination. Cardiovascular examination was normal without any murmurs. Lung auscultation was clear bilaterally. The abdomen was soft and non‐tender. Neurologically he was alert and oriented with no focal deficits or meningeal signs. The inguinal lymph nodes were palpable and tender bilaterally.

The most remarkable local finding was swelling of the left lower limb from the calf to the groin with mild erythema. On examination, the left leg was warm and tender with tense non‐pitting oedema. The left calf measured 19 in. around, while the right calf was 15 in. around. The distal pulses were intact in the affected limb. There were no blisters, necrotic changes or open wounds in the skin. Deep vein thrombosis, cellulitis and haematoma were included in the differential diagnoses at this stage. He was also noted to have exertional dyspnoea, but no orthopnoea and no chest pain. The detailed timeline of care for the patient is described in Table 1.

**TABLE 1:** Timeline of care.

19 Nov 2024 (Presentation and Admission)	Empiric treatment was started with Inf Linezolid 600 mg I/V BD, Inf Paracetamol 1 g I/V TDS, Inf N/Saline 500 mL I/V BD, Inj Omeprazole 40 mg I/V OD, and Inj Enoxaparin Sodium 60 mg S/C BD. Dengue serology drawn on the same day returned positive IgG and IgM antibodies with negative NS1 antigen, consistent with recent dengue infection.
20 Nov 2024	Lower‐limb Doppler ultrasound was negative for deep venous thrombosis; there was marked soft‐tissue edema of the left leg and tender bilateral inguinal lymph nodes, supporting the diagnosis of cellulitis. CT pulmonary angiography performed which ruled out pulmonary embolism but showed multiple bilateral pulmonary nodules, some cavitating and surrounded by ground‐glass attenuation, consistent with septic pulmonary emboli. A small left pleural effusion and subsegmental atelectasis of the left lower lobe were also noted. The patient was additionally started on Inj Piperacillin‐Tazobactam in adjunct to I/V Linezolid.
24 Nov 2024	Erythrocyte sedimentation rate (ESR) was 130 mm/h.
25 Nov 2024	Both transthoracic and transesophageal echocardiography showed biventricular function within normal limits and no valvular vegetations. They noted moderate mitral regurgitation, mild tricuspid regurgitation, and pulmonary hypertension. The patient developed acute onset of left‐sided chest pain which seemed of pleuritic nature and worsening dyspnoea. Chest radiography revealed a large left sided hydropneumothorax. The procalcitonin had decreased to 0.19 ng/mL. A left chest tube was inserted for the hydropneumothorax. The pleural fluid analysis was pale yellow exudative fluid with protein 3.26 g/dL, total cell count 1280/cmm, 80% neutrophils, and an ADA level of 60 U/L.
26 Nov 2024	Blood cultures showed no growth after 7 days of aerobic incubation. Serial complete blood counts demonstrated leukocytosis with neutrophilic predominance. The peak white blood cell count was 21.9 × 10^3^/μL.
29 Nov 2024	Chest tube was removed after complete chest expansion was observed on Chest X‐ray and there was no drain output for 48 h.
4 Dec 2024 (Discharge)	The patient's condition improved steadily over the following week. By 4 Dec 2024, swelling of the left leg had markedly subsided and no further fever spikes were documented. Repeat laboratory investigations showed a white blood cell count of 12.9 × 10^3^/μL and CRP of 3.29 mg/L. He was discharged home on oral Linezolid and Cefixime after removal of the chest tube.
11 Dec 2024 (Follow‐up)	On his first follow‐up visit after 1 week, patient had deteriorated with increased left leg swelling, high grade fever spikes up to 104 F documented. Chest X ray showed no evidence of hydropneumothorax. Patient was readmitted due to poor antibiotic adherence and recurrent symptoms, was administered Inj Vancomycin 1 g BD for 10 days during hospital stay with continuation of therapy advised for a total treatment duration of 21 days.

Abbreviations: ADA, Adenosine Deaminase; BD, Twice daily; CRP, C‐Reactive Protein; CT, Computed Tomography; IgG, Immunoglobulin G; IgM, Immunoglobulin M; Inf, Infusion; Inj, Injection; I/V, Intravenous; N/Saline, Normal Saline; NS1, Nonstructural protein 1 antigen; S/C, Subcutaneous; TDS, Three times daily.

## 4 Diagnostic Assessment

On admission, laboratory testing showed hemoglobin 11.1 g/dL, white blood cell count 9.0 × 10^9/L, and platelets 242 × 10^9/L. Inflammatory markers were markedly elevated, with procalcitonin 18.14 ng/mL, C‐reactive protein 412 mg/L, and erythrocyte sedimentation rate 130 mm/h. Renal and hepatic tests showed creatinine 1.91 mg/dL and alanine aminotransferase 66.1 U/L, while coagulation profile and serum electrolytes were normal. Peripheral smear was normocytic and normochromic, lactate dehydrogenase was 741 U/L, and reticulocyte count was 1%.

Microbiological investigations did not identify a causative organism. Dengue serology was positive for IgM and IgG antibodies, while dengue NS1 antigen was negative. Blood cultures showed no growth after 7 days of aerobic incubation. The patient admitted to consuming antibiotics earlier to presenting to the hospital. HIV serology was non‐reactive. Doppler ultrasound of both lower limbs demonstrated soft‐tissue swelling of the left leg with bilateral enlarged inguinal lymph nodes but no evidence of deep venous thrombosis.

**FIGURE 1:**
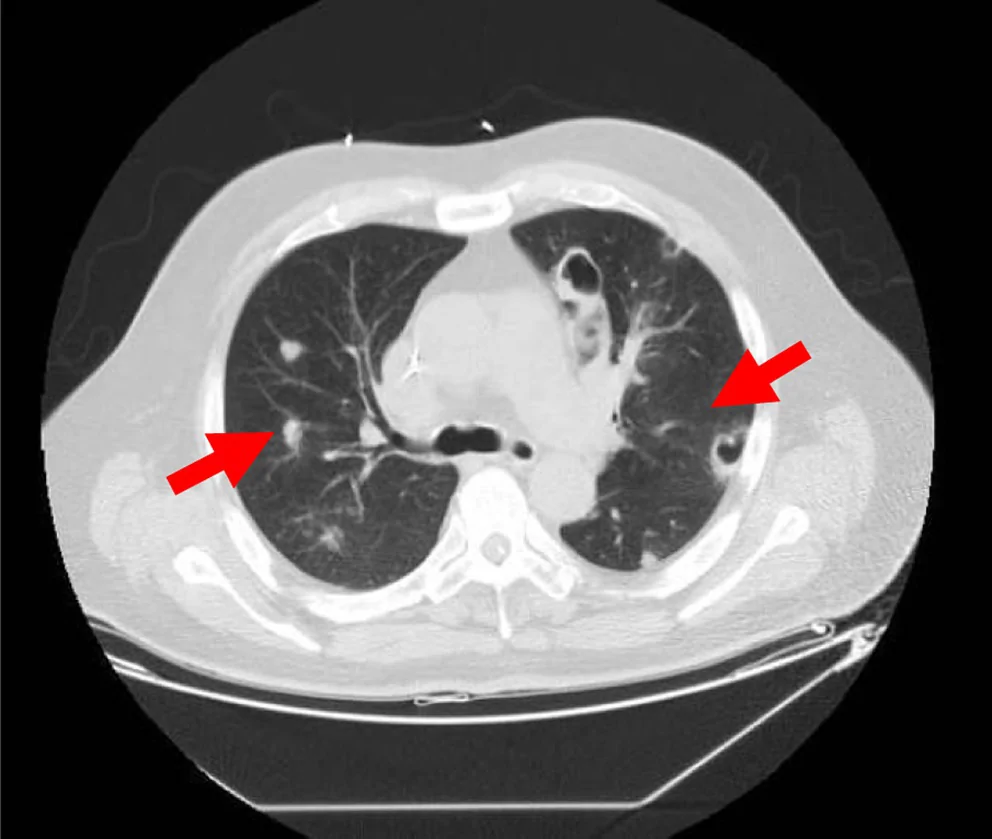
There are multiple irregular nodules in the right lung; there is a subpleural nodule with cavitation and feeding vessel sign.

**FIGURE 2:**
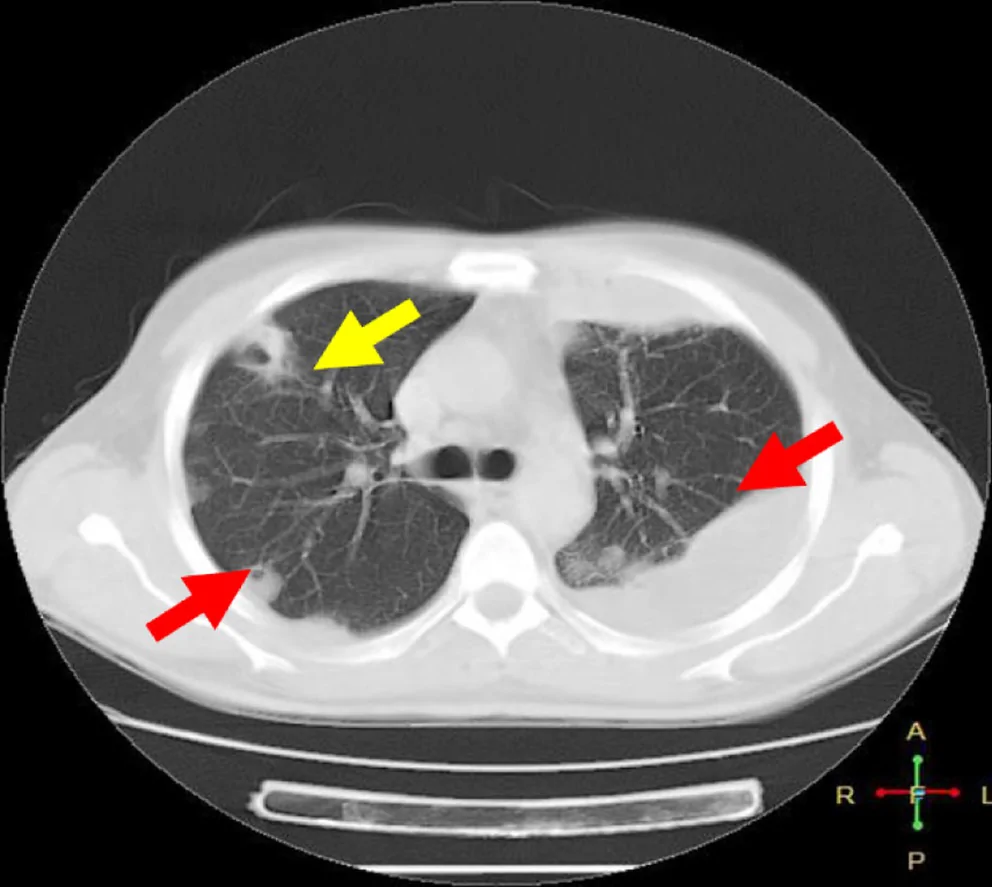
Multiple small cavitating pulmonary nodules that display typical feeding vessel sign.

CT pulmonary angiography demonstrated no thrombus within the pulmonary trunk, right or left pulmonary arteries, or their segmental branches. However, both lungs showed multiple bilateral cavitating and non‐cavitating nodules of varying sizes, shown in Figures 1 and 2. Some of these were associated with surrounding ground‐glass haze, along with subsegmental atelectatic changes in the posterior basal segment of the left lower lobe, and mild left pleural thickening with effusion. These findings were more consistent with septic pulmonary embolism than thrombotic pulmonary embolism.

Infective endocarditis was considered because of pulmonary lesions but was excluded by negative transthoracic and transesophageal echocardiographic findings. Moderate mitral regurgitation, mild tricuspid regurgitation, and pulmonary hypertension were noted, but no valvular vegetations were seen. Other differential diagnoses considered included cellulitis without embolization and hematoma of the left leg.

Pleuropulmonary tuberculosis was also considered because of the family history of pulmonary tuberculosis. However, the overall clinical picture was not typical of tuberculosis. The illness had an acute onset over only a few days, with rapidly progressive lower‐limb cellulitis and markedly elevated inflammatory markers suggesting an acute bacterial infection. Later pleural fluid analysis demonstrated a neutrophil‐predominant exudate (80% neutrophils), which was less supportive of tuberculous pleuritis, and no microbiological evidence of tuberculosis was identified during the hospital stay.

**FIGURE 3:**
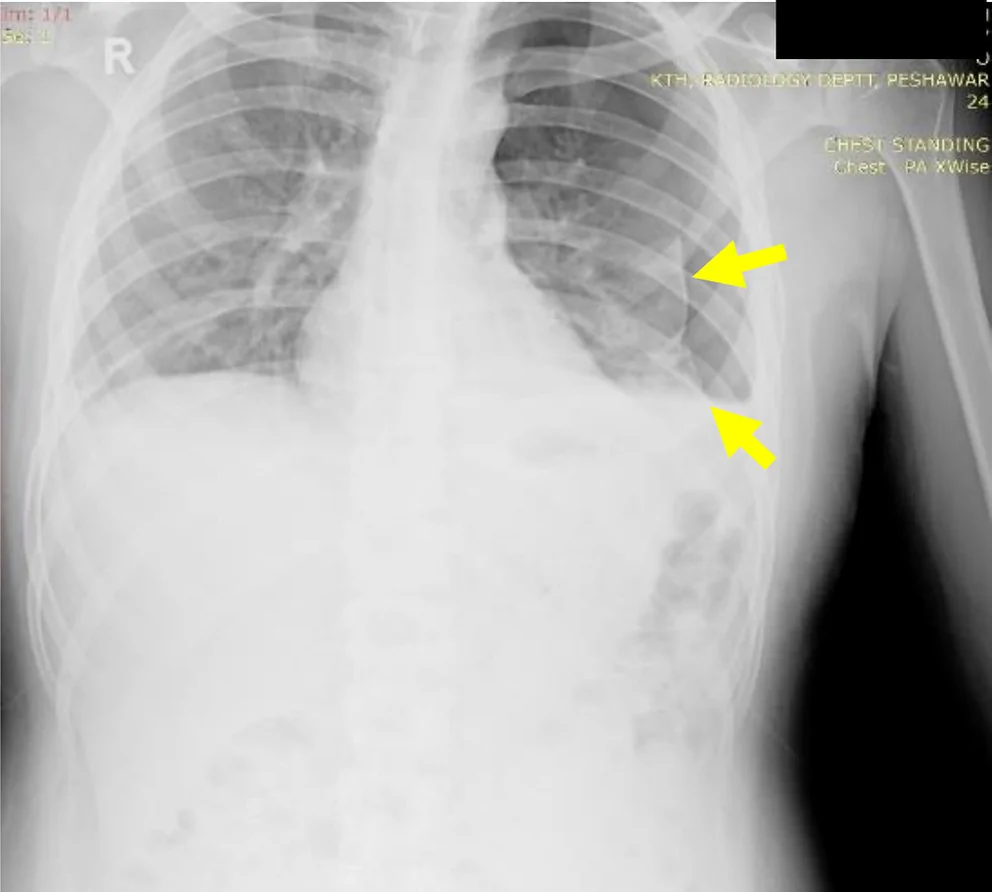
Left‐sided Hydro‐pneumothorax.

During hospitalization, the patient developed sudden‐onset pleuritic chest pain and worsening dyspnea. On examination, breath sounds were reduced and percussion was dull on the left lung base. Chest radiography demonstrated a left‐sided hydropneumothorax shown in Figure 3.

Pleural fluid analysis fulfilled Light's criteria for exudative effusion, with pleural fluid protein 3.26 g/dL, pleural fluid LDH 2626 U/L, pleural fluid‐to‐serum protein ratio 0.50, and pleural fluid‐to‐serum LDH ratio 3.54. Microscopy showed a total cell count of 1280/cmm with predominant neutrophilia (80%), while ADA level was 60 U/L. Gram stain and culture of the pleural fluid were not performed.

The final diagnosis was culture‐negative septic pulmonary embolism, most likely secondary to left lower‐limb cellulitis following recent dengue fever, complicated by left‐sided hydropneumothorax.

## 5 Therapeutic Intervention

The patient was initially managed with intravenous antibiotics and supportive treatment. Pharmacological therapy included linezolid 600 mg intravenously twice daily, paracetamol 1 g intravenously three times daily, omeprazole 40 mg intravenously once daily, and enoxaparin 60 mg subcutaneously twice daily. Intravenous normal saline 500 mL twice daily was administered as supportive therapy. During hospitalization, piperacillin‐tazobactam 4.5 g intravenously three times daily was added. Dexamethasone, ipratropium nebulization, and serratiopeptidase were also given in response to the evolving clinical condition. Anticoagulation with enoxaparin was continued.

**FIGURE 4:**
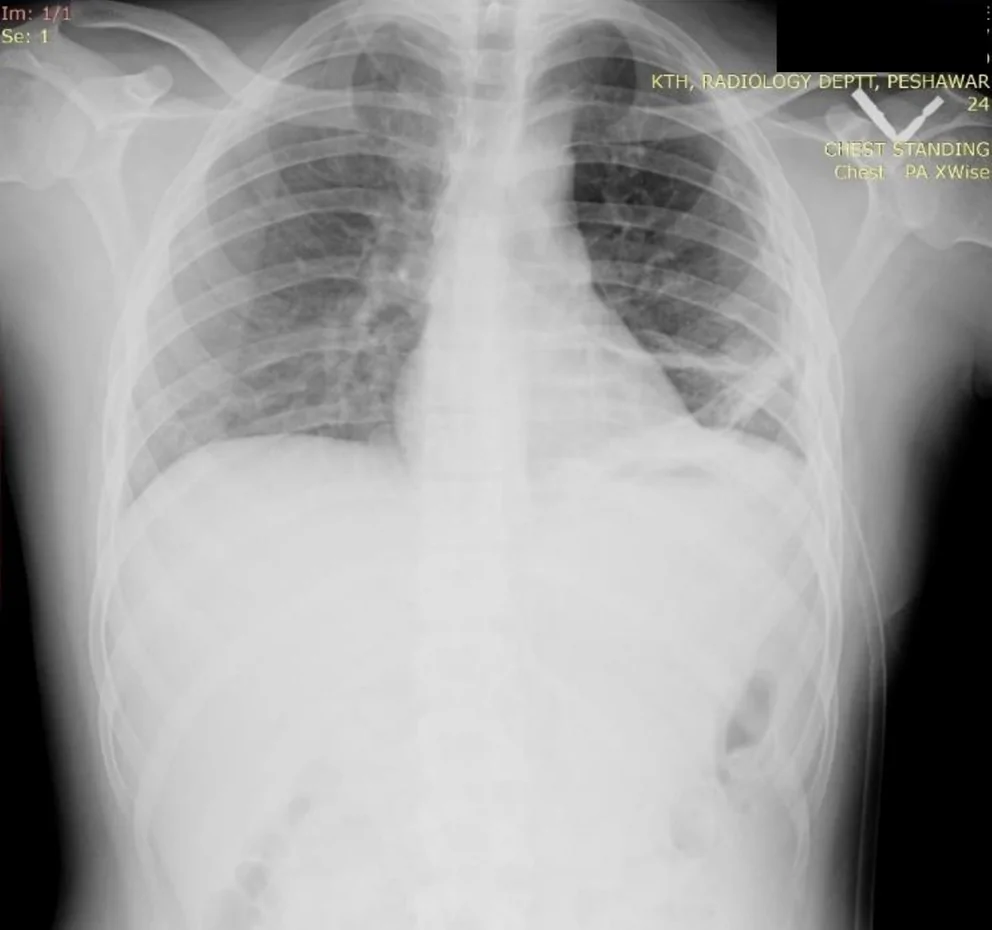
Chest radiograph following tube thoracostomy.

Following the development of left‐sided hydropneumothorax, tube thoracostomy was performed (shown in Figure 4). The chest tube was removed after 5 days when complete lung re‐expansion was confirmed on chest radiography, and no drain output had been observed for 48 h. The patient was discharged on oral linezolid and cefixime.

## 6 Follow‐Up and Outcomes

During hospitalization, the patient demonstrated progressive clinical and laboratory improvement following broad‐spectrum intravenous antibiotics, anticoagulation, and supportive care. No further fever spikes were documented, and the left‐leg swelling gradually resolved. By 4 December 2024, the white blood cell count had decreased to 12.9 × 10^3^/μL and CRP to 3.29 mg/L, while procalcitonin had fallen to 0.19 ng/mL on 27 November 2024. These changes correlated with improvement in dyspnoea and left‐leg cellulitis.

The principal complication during hospitalization was a left‐sided hydropneumothorax managed with left tube thoracostomy (chest‐drain insertion). Pleural fluid analysis was limited to biochemical and cytological studies; Gram stain and bacterial culture were not performed on the pleural fluid, precluding microbiological correlation with the pulmonary lesions. Blood cultures remained negative throughout the admission. No bleeding complications related to anticoagulation, antibiotic‐related adverse reactions, or other treatment‐associated toxicities were documented in the available records. At discharge, the patient was afebrile with marked improvement in left lower‐limb cellulitis.

On his first follow‐up visit after 1 week, he seemed to have deteriorated, with increased left leg swelling and high‐grade fever spikes up to 104 F documented. His repeat chest X‐ray showed no evidence of any hydropneumothorax. On inquiry, he had not been compliant with his antibiotic regimen. The patient was readmitted and administered intravenous vancomycin 1 g twice daily for 10 days during hospital stay and was advised the same for home treatment once improved for a total duration of 21 days.

## 7 Discussion

SPE frequently presents with nonspecific symptoms. In the systematic review by Ye et al., fever was reported in 144 of 168 cases, dyspnoea in 81, chest pain in 82, and cough in 69; chest computed tomography most commonly demonstrated multiple peripheral pulmonary nodules, which were reported in 89 cases, while cavitation and pleural effusion were identified in 75 and 40 cases, respectively [2]. In contrast to the typical presentation, our patient was evaluated during the recovery phase of dengue fever, was afebrile on admission, and presented primarily with unilateral lower‐limb cellulitis and exertional dyspnoea. In this setting, the coexistence of soft‐tissue infection and respiratory symptoms required careful distinction between thrombotic pulmonary embolism and SPE. Lower‐limb Doppler ultrasonography demonstrated no deep venous thrombosis, and CT pulmonary angiography showed no pulmonary arterial filling defects. The presence of multiple bilateral peripheral pulmonary nodules, several of which were cavitary, was therefore most consistent with SPE [2].

The temporal sequence observed in this patient—recent dengue infection, followed by lower‐limb cellulitis, then septic pulmonary embolism, and subsequently hydropneumothorax—is clinically compelling but does not, in itself, establish a causal chain; each link is presented here as a plausible clinical association rather than a proven mechanism. Recent dengue infection may have contributed to susceptibility to secondary bacterial infection. Trunfio et al. reported that concurrent bacteremia complicates 0.18%–7.0% of dengue infections and highlighted that persistent fever together with abnormalities in inflammatory markers should raise suspicion for bacterial coinfection [6]. In the present case, inflammatory markers were markedly elevated, with a procalcitonin level of 18.14 ng/mL, CRP of 412 mg/L, and ESR of 130 mm/h. These abnormalities subsequently improved in parallel with clinical recovery, with procalcitonin decreasing to 0.19 ng/mL and CRP to 3.29 mg/L. While these findings support the presence of a significant bacterial inflammatory process, they do not establish the identity of the causative organism. Proposed mechanisms for bacterial superinfection in dengue include transient alterations in host immune function and disruption of endothelial and mucosal barriers [6, 7]. Although these mechanisms provide a biologically plausible explanation for the sequence observed in our patient, a direct causal relationship cannot be established from a single case.

The microbiological and cardiac evaluations did not identify a definitive source of embolization. Blood cultures remained negative, and both transthoracic and transoesophageal echocardiography demonstrated no valvular vegetations, arguing against infective endocarditis as the source of septic emboli. However, blood cultures were obtained after the patient had already initiated antimicrobial therapy, which may have reduced microbiological yield. Because pleural fluid culture results were not available and no organism was isolated, the diagnosis should be regarded as culture‐negative SPE occurring in association with lower‐limb cellulitis following recent dengue fever. Although *Staphylococcus aureus* is among the most frequently reported pathogens in SPE, [2] the available data in this case do not permit attribution to any specific organism. Culture‐negative SPE of this kind is a recognized, if less common, presentation of the condition; in one comparative series, 4 of 10 SPE cases were culture‐negative, with a shorter and less severe clinical course than culture‐positive cases [8].

The empirical antimicrobial regimen (linezolid, with piperacillin‐tazobactam added) was selected in keeping with published guidance for severe cellulitis with systemic involvement of uncertain microbiology, which recommends broad empirical coverage—such as vancomycin or linezolid combined with piperacillin‐tazobactam or a carbapenem—to address both polymicrobial and monomicrobial (including methicillin‐resistant *Staphylococcus aureus* ) etiologies [9]. This combination also provided coverage against *Staphylococcus aureus* , the organism most frequently implicated in septic pulmonary embolism [2]. As no organism was ultimately isolated, therapy could not be de‐escalated or tailored on microbiological grounds. Antibiotic duration was instead guided by clinical response—defervescence, resolution of leukocytosis, and decline in procalcitonin and CRP—broadly consistent with the extended courses, commonly 4–9 weeks, reported for SPE [2]. The relapse that occurred after discharge, attributable to documented non‐adherence with oral therapy, further illustrates the importance of completing the intended course and accounts for the eventual cumulative duration of antimicrobial therapy in this patient.

Enoxaparin 60 mg subcutaneously twice daily was commenced on the day of admission, in the setting of markedly asymmetric lower‐limb swelling with exertional dyspnoea—a presentation carrying a reasonable pretest probability for deep venous thrombosis with pulmonary embolism, for which empirical anticoagulation pending confirmatory imaging is standard practice. Lower‐limb Doppler ultrasonography and CT pulmonary angiography subsequently excluded venous and arterial thrombus, respectively, and the diagnosis was revised to septic pulmonary embolism, a condition for which anticoagulation is not routinely recommended in the absence of an independently demonstrated venous thrombus. The specific documented rationale for continuing anticoagulation beyond this point is not available in the retrospective case records; we acknowledge this explicitly and present its continuation as a point of clinical judgment in an evolving diagnostic picture, rather than as a protocol‐directed or fully reconstructable indication.

The subsequent development of a left hydropneumothorax represents an additional noteworthy feature of this case. The hydropneumothorax occurred after pulmonary lesions consistent with SPE had already been identified, and pleural fluid analysis demonstrated a neutrophil‐predominant exudative effusion. We propose, as a hypothesis rather than an established mechanism, that extension or rupture of a cavitary septic pulmonary lesion into the pleural space—a recognized, if uncommon, complication of cavitary or necrotic pulmonary infections [10]—may explain this event. However, no follow‐up thoracic imaging, demonstration of a bronchopleural fistula, or pleural fluid Gram stain/culture was available to confirm a shared source between the pulmonary and pleural findings in this patient, and this proposed mechanism should therefore be regarded as hypothetical rather than established.

The strengths of this report include detailed chronological documentation, comprehensive radiological evaluation with Doppler ultrasonography and CT pulmonary angiography, exclusion of cardiac vegetations through transthoracic and transesophageal echocardiography, and serial monitoring of inflammatory markers demonstrating objective response to treatment.

Several limitations should also be acknowledged. No causative organism was identified; pleural fluid Gram stain and culture were not performed; and blood cultures were obtained after antimicrobial therapy had been initiated. The specific rationale documented at the time for continuing anticoagulation once venous and arterial thrombosis had been excluded could not be established from the retrospective case records. These limitations restrict the certainty with which the underlying microbiology, embolic source, and management decisions in this case can be interpreted.

## 8 Conclusion

This case highlights the importance of considering secondary bacterial infection in patients with recent dengue fever who develop new focal infections, markedly elevated inflammatory markers, or respiratory symptoms that are not readily explained by uncomplicated dengue. When pulmonary imaging demonstrates multiple peripheral cavitary nodules in this setting, SPE should be included in the differential diagnosis even when blood cultures are negative and thrombotic pulmonary embolism has been excluded. Early recognition and prompt treatment may help prevent further complications and contribute to favorable clinical outcomes.

## Author Contributions

**Faiza Arif:** conceptualization, writing – review and editing, resources. **Mariyah Saqib Qureshi:** conceptualization, supervision, writing – review and editing. **Sajjad Khan:** investigation, validation, writing – original draft. **Malaika Usman:** writing – original draft, writing – review and editing. **Umama Rehman:** investigation, project administration, writing – original draft. **Aiyna Usman:** resources, writing – original draft. **Bilal Wazir Khan:** writing – original draft, writing – review and editing. **Kashif Yousaf:** project administration, resources, writing – review and editing.

## Funding

The authors have nothing to report.

## Consent

Written informed consent was obtained from the patient for publication of this case report and accompanying images.

## Conflicts of Interest

The authors declare no conflicts of interest.

## Data Availability

Data sharing not applicable to this article as no datasets were generated or analyzed during the current study.
